# Barriers and facilitators of shared decision making in acutely ill inpatients with schizophrenia—Qualitative findings from the intervention group of a randomised‐controlled trial

**DOI:** 10.1111/hex.13313

**Published:** 2021-07-13

**Authors:** Stefanie Becher, Fabian Holzhüter, Stephan Heres, Johannes Hamann

**Affiliations:** ^1^ Klinik und Poliklinik für Psychiatrie und Psychotherapie Technische Universität München Munich Germany; ^2^ Isar‐Amper‐Klinikum München Nord Munich Germany

**Keywords:** health services research, patient autonomy, schizophrenia, shared decision making

## Abstract

**Background:**

Shared decision making (SDM) is appreciated as a promising model of communication between clinicians and patients. However, in acute mental health settings, its implementation is still unsatisfactory.

**Objective:**

The aim of this study is to examine barriers and facilitators of SDM with acutely ill inpatients with schizophrenia.

**Design:**

A qualitative interview study was performed.

**Setting and Participants:**

The analysis is based on interviews with participants (patients and staff members) of the intervention group of the randomised‐controlled SDM^PLUS^ trial that demonstrated a significant improvement of SDM measures for patients with schizophrenia on acute psychiatric wards.

**Main Variables Studied:**

Interviews addressed treatment decisions made during the current inpatient stay. The interviews were analysed using qualitative content analysis.

**Results:**

A total of 40 interviews were analysed and 131 treatment decisions were identified. According to the interviewees, SDM had taken place in 29% of the decisions, whereas 59% of the decisions were made without SDM. In 16%, a clear judgement could not be made. Barriers and facilitators of SDM were categorised into patient factors, clinician factors, setting factors and others. Clinicians mostly reported patient factors (e.g., symptoms) as barriers towards SDM, which were not mirrored on the patients' side. Facilitators included patient as well as clinician behaviour during consultations.

**Conclusion:**

Even in the context of a successful SDM intervention, the implementation of SDM for patients in the very acute stages of schizophrenia is often not possible. However, strong facilitators for SDM have also been identified, which should be used for further implementation of SDM.

**Patient or Public Contribution:**

During the development of the study protocol, meetings with user representatives were held.

## 1 INTRODUCTION

Some stakeholders propose shared decision making (SDM) to be the gold standard of patient–physician decision making, including mental health settings.^1^, ^2^ Others are more sceptical and highlight that patients' preferences for participation ‘differ associated with their personality, background and experiences, and may vary even for the same patient depending on the given health problem, the context, the specific content of the consultation, and the mood on the day’.^3^ Despite these concerns, it is widely accepted that ‘SDM has the potential to contribute to supporting people to live as well as possible in communities of their own choosing’.^4^

However, psychiatrists still hesitate to implement SDM for more acutely ill patients,^5^ and there is evidence that the implementation of SDM in acute psychiatric inpatient care faces specific challenges, particularly communication between patients and providers,^6^ which is a central aspect of SDM. To account for these special demands, adaptations to SDM have been developed to better address acutely ill patients and their clinicians, including the SDM^PLUS^ approach.^7^ SDM^PLUS^ offers specific communication techniques for patients (e.g., social skills training) and therapists (e.g., motivational interviewing) to support more effective decisional discussion, even in an acute mental health care setting.

SDM^PLUS^ has recently been evaluated in a large cluster‐randomized controlled trial, which has shown that the intervention was superior in comparison to the standard treatment with regard to patients' perceived involvement in decision making, their treatment satisfaction and the therapeutic alliance.^8^

In the present paper, we present qualitative data gathered from patients and physicians from the intervention group of the SDM^PLUS^ trial. The aim of this analysis is to take a closer look at decision‐making patterns as well as at barriers and facilitators of decision making within the context of an ‘effective’ SDM intervention.

## 2 METHODS

### 2.1 Participants

Participants of this qualitative study were from the intervention group of the SDM^PLUS^ trial. This prospective, cluster‐randomised, mixed‐methods trial took place in 12 acute psychiatric wards from five participating hospitals in Germany. In total, 322 patients aged 18–65 years with a diagnosis of schizophrenia or schizoaffective disorder were recruited. The intervention consisted of a staff training group and a patient training group. Staff (including residents, consultants, psychologists and nurses) from intervention wards participated in two interactive, half‐day workshop sessions about the communication techniques of SDM^PLUS^. Patients of the intervention group took part in group trainings on SDM twice a week.^9^ Patients and staff in the control group received treatment as usual (TAU). The quantitative results of the study have been reported elsewhere.^8^

The interviewers (S. B. and F. H.) were also involved in patient recruitment and the provision of the intervention, and they were already familiar with the patients and staff. Following the principles of *purposeful sampling*, they personally addressed patients/staff members whenever there was any hint of either particularly high or low implementation of SDM and tried to recruit them for the qualitative interviews.

### 2.2 Data acquisition and analysis

Face‐to‐face interviews were conducted with all participants using a topic guide (see Supporting Information Appendix), which had been developed by the whole research group and pretested on the participating wards. Topics addressed were patients' general treatment satisfaction, their level of participation and how this had potentially changed during the course of the inpatient stay. Further, patients were asked about their experiences with physician consultations on the wards, with a special focus on what individual decisions were about and how they were initiated, discussed and made. Patients were encouraged to present specific decisions and were inquired to provide further details on who was involved and whether or not decisions were shared. Finally, experiences with study‐related interventions (patient groups, staff training) as well as experiences with coercion or regulations on the wards were queried. For the clinicians' interviews, an adaption of this topic guide was used.

All interviews were conducted by S. B., at that time a medical student, and F. H., MD. Both researchers were trained and supervised by J. H. in qualitative methods; during the interviews, no other persons were present. Interviews were audio‐recorded and transcribed verbatim. Two researchers coded all of the transcripts. Here, S. B. and F. H. began by individually coding approximately 20% of the data. Then, the results were compared and contrasted, thus adjusting and unifying the coding method.

In a second step, S. B. then went on to code the entire material accordingly. F. H. then independently coded data extracts and compared them to the individual findings of S. B. This was followed by a third rundown through the material by S. B. J. H. acted as a consultant on particularly difficult coding decisions. We used MAXQDA12 software for the qualitative data analysis and followed the principles of content analysis.^10^

**Figure 1:**
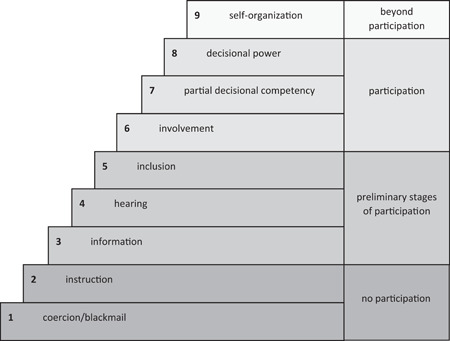
Level model of participation

The analysis followed a multistep approach. First, treatment decisions were identified from the transcripts and then categorised as to whether SDM was present or not. As the classification method according to ‘steps of SDM’^11^ proved to be incompatible with participant responses, we applied the *level model of participation* created by *Wright* ^12^ to the data. This 9‐level model is derived from health promotion research and is designed to measure participation regarding health advancement and prevention in communities, and later utilised by participatory health researchers. For this study, the model was adjusted to fit the different aspects of patient–clinician consultations (Figure 1). According to this model, genuine participation starts at Level 6, whereas lower categories are considered as preliminary stages of participation (3–5) or no participation (1–2). Level 9 extends beyond participation. Decisions described by participants were then coded using the adjusted level model. Because of the distribution of codings (see Section Design, 3), we decided to dichotomise our codings into SDM (levels 6–9) and no SDM (levels 1–5), even if level 9 (‘self‐organization’) rather overlaps with the informed decision making model,^13^, ^14^ indicating even greater patient autonomy as suggested for SDM.

In the second step of the analysis, confounding factors (facilitators and barriers) were inductively developed from the material. Coding took place for all decisions that had been identified and dichotomised in SDM and no SDM in Step 1.

This trial, which included the qualitative interviews, had been approved by the local review board (Ethikkommission der Technischen Universität München).

## 3 RESULTS

The analysis was based on 40 interviews with 18 patients and 14 different corresponding clinicians. In 14 cases, patient interviews were matched by one corresponding clinician interview. In two cases, a patient interview was matched by two or three corresponding clinician interviews. In two cases, two interviews with the same patient were conducted at different stages of the treatment. One interview was held with two corresponding clinicians (who were both involved in the patient's care) at the same time. Interview length varied from 5 to 22 min. Table 1 shows the demographic and clinical characteristics of the participants. Eleven out of Eighteen patients had been admitted involuntarily to the hospital. None of the patients of the initial trial refused to participate in the qualitative interviews.

**Table 1:** Characteristics of participants

Patients	Gender	Age	Diagnosis
LMK19002	F	19	Paranoid schizophrenia
LMK19009	M	62	Paranoid schizophrenia
LMK19011	F	44	Paranoid schizophrenia
LMK19014	F	58	Paranoid schizophrenia
LMK19016	M	57	Schizoaffective disorder
LMK19017	F	42	Paranoid schizophrenia
IAKP1026	M	38	Paranoid schizophrenia
IAK28025	F	41	Paranoid schizophrenia
IAKBE007	F	59	Paranoid schizophrenia
IAKBE013	F	51	Paranoid schizophrenia
IAKBE015	F	38	Paranoid schizophrenia
IAKBE018	M	33	Paranoid schizophrenia
IAKAE018	F	35	Paranoid schizophrenia
BKHC2003	M	37	Paranoid schizophrenia
BKHC2010	F	18	Hebephrenia
BKHC2018	M	20	Hebephrenia
BKHC2014	F	28	Schizoaffective disorder
BKHE2018	M	65	Paranoid schizophrenia

**Clinicians**	**Gender**	**Age group**	**Professional background**
LMK19002‐dr/LMK19016‐dr	F	30–35	Neurology resident
LMK19014‐dr	F	25–30	Psychiatry resident
IAKP1026‐dr	F	30–35	Psychiatry resident
IAK28025‐dr	F	40–45	Psychiatry resident
IAKBE007‐dr/IAKBE013‐dr/IAKBE015‐dr	M	35–40	Psychiatry resident
IAKBE018‐dr	F	30–35	Psychiatry resident
IAKAE018‐dr	F	45–50	Psychiatry resident
BKHC2003‐dr	M	30–35	Psychiatry resident
BKHC2010‐psy	F	40–45	Psychologist
BKHC2018‐dr	M	30–35	Psychosomatic Medicine Resident
BKHC2018‐nu	F	35–40	Nurse
BKHC2014‐dr‐ow/BKHE2018‐dr	F	25–30	Psychiatry resident
BKHC2014‐dr‐cw	F	25–30	Neurology resident
BKHC2014‐psy	F	25–30	Psychologist

Abbreviations: F, female; M, male.

### 3.1 Treatment decisions and stages of participation

One hundred and thirty‐one treatment decisions were identified from the interviews. The topic of *drug decisions* was the most frequently named category (*n* = 75). Other decision topics included *leave from ward* (*n* = 21), *transfer to an open ward* (*n* = 7), *length of stay* (*n* = 7), *arrangements for the time after discharge* (*n* = 18) and *others* (*n* = 3).

**Figure 2:**
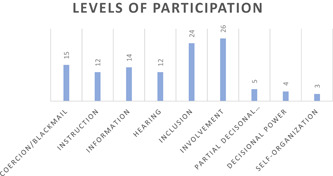
Levels of participation

We were able to assign 115 decisions (88%) to the *level model of participation*. As can be seen in Figure 2, only 38 decisions (29%) were assigned to Levels 6 and higher, whereas 77 decisions (59%) were assigned to Levels 1 through 5. The remaining 16 decisions (12%) were unassignable due to unclear wording from interviewees (Figure 2).

Patients responses indicated a lower mean achieved level of participation (*M* = 4.0) as compared to clinicians (*M* = 4.5).

Table 2 shows the quotes extracted from the material typical for the different levels. Thus, all stages of decision making were present in the data set, including coercion/blackmail, which was the third most commonly assigned code.

**Table 2:** Coding representatives for the level model

Level Nr	Level of participation	Quote
1	Coercion	Doctor: Initially [the patient] refused any medication aside from *quetiapine*. But then, after her leave agreement was cancelled, she was willing to accept medication and agreed to taking *amisulpride. (IAKBE015‐dr, section 14)*
2	Instruction	Doctor: The patient does do what he is being told to do, but really, he has no real interest in any of it. Therefore, one can really only tell him: ‘*Take this medication now!’* and he will then take it, but we were not successful in creating any sort of understanding within him. *(BKHC2003‐dr, section 11)*
3	Information	Patient: [The doctor] told me: *‘Now we are going to try this medication’. (BKHE2018, section 31)*
4	Hearing	Doctor: The patient described the side‐effects that had occurred and we reacted accordingly. *(IAKBE007‐dr, section 13)*
5	Inclusion	Patient: Here on this ward the doctors very much looked after us patients, told us how to deal with the medication, and also the side effects. For example, I had this restlessness in my legs and the doctor then did lower the medication a bit, so that this would go away. *(IAKBE013, Sections 5–7)*
6	Involvement	Doctor: We thought about the medication to which we could switch and the patient agreed to taking *risperidone*. She wanted something that didn't cause significant weight gain, therefore *olanzapine* for example would not have been an option for her. (IAK28025‐dr, section 9)
7	Partial decisional competency	Doctor: Concerning the medication, well, he hasn't been taking anything for a while now, ever since he discarded the last medication, *Abilify*. And we then let things run their course on a trial basis. Since nothing changed clinically, we didn't see any reason to motivate him for anything from that point on. *(BKHC2018‐doctor, section 11)*
8	Decisional power	Doctor: Ultimately, what will happen after his stay in the hospital will be decided more by him [than us]. *(BKHC2018‐doctor, section 25)*
9	Self‐organisation	Patient: And now I found myself an apartment through the housing office. I got myself the authorization note for that, despite being under legal guardianship. I chose the nursing service and this is all going to be set up now. *(IAKAE018, section 43)*

### 3.2 Barriers and facilitators of SDM

**Figure 3:**
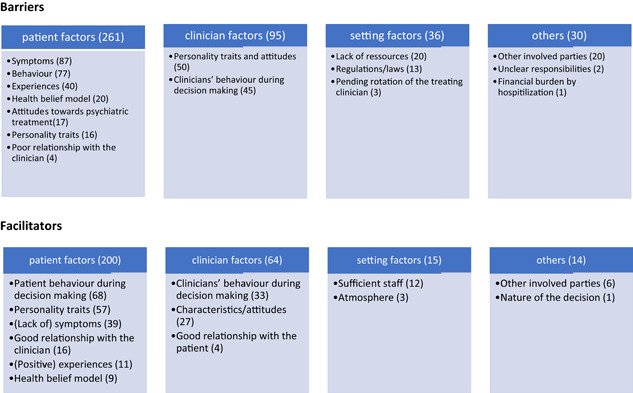
Barriers and facilitators of shared decision making

To classify barriers or facilitating factors of SDM, categories were developed inductively. These were later assigned to the overarching categories *factors facilitating SDM* and *factors hindering SDM* as well as to the intermediate categories *patient‐related factors*, *clinician‐related factors*, *setting‐related factors* and *other factors*. The final distribution of categories is displayed in Figure 3.

In total, 59% of the codes were assigned to the *barriers*, whereas 41% were assigned to the *facilitators*. Approximately two‐thirds of codes (both for barriers and facilitators) were assigned to *patient‐related factors*, followed by approximately 20% *to clinician‐related factors. Setting factors* and *other factors* added less than 10% each.

Most *patient‐related factors* were derived from clinicians' statements. Among *clinician‐related factors*, a slight majority originated from patients' descriptions. Among different categories, codes from both parties were more or less balanced.

#### 3.2.1 Barriers towards SDM—patient‐related factors

*Symptoms* (*n* = 87) were cited as the most common barrier in both patient and clinician interviews, which could be further classified into impaired decisional capacity, impaired communication and, in the case of extreme symptoms, limited/restricted treatment options. Decisional capacity was described as impaired due to lack of reflection, thought disorder, ambivalence or lack of ability to plan ahead (‘*I think the biggest barrier with her is her complete and utter lack of suffering, there just isn't any. She also doesn't realize that she creates suffering in others, in her environment. She simply isn't accessible to any rational discussion’ (LMK19014‐dr)*. Delusions and hallucinations were rarely reported as a barrier towards SDM. Communication was reported to be impaired by depressive symptoms, marked anxiety, mutism or logorrhoea. In some cases, patient symptoms were severe enough that clinicians felt limited in their ability to offer different treatment options to patients. Limitations due to patient symptoms were reported more often by clinicians than by patients, which is illustrated by two opposing perspectives on the same situation (corresponding clinician–patient pair):

*P: ‘Doctor's consultations went pretty well. I was always able to connect fairly well [with the clinicians], was always able to present myself well and get across my issues’. ([AKBE018)*

*D: ‘… when he first arrived at the facility, he was very hard to reach, he didn't want to take any medication, he didn't want to cooperate at all, and he was all caught up in his delusional structure of thinking’. (IAKBE018‐dr)*

The second most commonly named barrier among patient‐related factors was *behaviour* (*n* = 77), which included blind trust, passivity, manipulation and overexcitement, as well as lack of motivation, knowledge and openness towards clinicians' suggestions (*‘What makes it difficult is that she is very hostile to whatever we would propose to her […] and, basically, she pretty much only does whatever she thinks is right’. [LMK19002‐dr]*). Also, certain behaviours that occurred during inpatient stay were regarded to impede SDM, such as abusive behaviour, lawsuits against hospital staff, violation of rules and deception.

The category *experiences* (*n* = 40) was composed of mostly negative experiences in terms of medication, staff or psychiatric facilities, as well as previous experiences of powerlessness (*‘They were very clear through their body language that they didn't want to hear any more objections at that point and that it was just going to be their way now. They know all too well that they have the power here’. [IAKBE015]*)

Patients' *health belief model* (*n* = 20) was a barrier predominantly named by clinicians. This included a lack of acceptance of the disease or insight, a lack of understanding thereof and the conviction that medication is not required for treatment. *Attitudes towards psychiatric treatment* (*n* = 17) included patients' general negative attitudes towards psychiatric inpatient treatment and medication. Certain patient *personality traits* (*n* = 16) were further reported to handicap SDM, such as aggression and conformity. Some patients handed over responsibility for treatment decisions entirely to the clinician. A general lack of patient interest in participation was rarely reported. Lastly, *poor relationship with the clinician* was occasionally stated to be a hindering factor, mostly due to lack of trust in the clinician.

#### 3.2.2 Barriers towards SDM—clinician‐related factors

*Personality traits and attitudes* (*n* = 50) were the most commonly reported clinician‐related factors impeding SDM. This included attitudes towards SDM and towards the patients themselves. SDM was generally ruled out by some clinicians in certain situations (e.g., in potentially critical circumstances and in highly symptomatic patients). Others did not regard SDM as necessary or helpful for certain ‘types’ of patients. Some clinicians' attitudes towards patients could in fact impede SDM, including, for example, lack of openness towards the patient's view or lack of appreciation for a patient's attempt at engagement.

*Clinicians' behaviour during decision making* (*n* = 45) was repeatedly perceived to be problematic. Listed behaviours included not addressing patient wishes, not being able to let go of one's own ideas, restricting treatment possibilities to one option or even applying pressure to force acceptance of treatment (*Physician: ‘… she categorically refused [medication], but after her leave was called off, she consented to medication and was then willing to take amisulpride’. (IAKBE015‐dr)*

#### 3.2.3 Barriers towards SDM—setting‐related factors and others

Setting‐related factors that impeded SDM included lack of resources (staff, hospital beds, availability of various therapy programmes on the wards), court orders, laws or regulations and the pending rotation of the treating clinician. Other factors included further involved parties (nursing staff, legal guardians, other patients and family members).

#### 3.2.4 Facilitators of SDM—patient‐related factors

*Patient behaviour during decision making* (*n* = 68) was the most commonly cited facilitator among patient factors. This category consisted of the subcategories open‐mindedness for the clinicians' views or suggestions, active communication and preparation for consultations. Clinicians considered patient openness to be of great importance: *Physician: ‘The patient himself proved to be very open‐minded and he is going to take part in the decision, if he actually wants to add some further medication or… we're just going to have to discuss it together, yes’. (LMK19016‐dr)*

Active communication by the patient was also reported to be an important tool, since it involved the patient actively taking part in the decisional conversations (expressing wishes or fears, asking questions, giving feedback). *Physician: ‘… so with him, his worst fear was weight gain. This didn't occur in the end, so we have actually found the right medication for him now with* amisuplride’. *(IAKBE018‐dr)*

By communicating actively, patients were able to partly steer decisions themselves. One patient reported active communication to have been vital for participation, as it forced clinicians to engage more deeply in the decision‐making process. Good preparation was largely achieved through the participation in patient groups in the context of the intervention but also through self‐study, psychoeducation in group sessions and also individually by the clinicians.

Patients' *personality traits* were considered to be another important facilitator for SDM. These included different clusters such as engagement and assertiveness (*‘I think I am someone who talks a lot during a doctor's consultation and who says what he wants. And they [the clinicians] went along with that. […] So in a way they were forced to react’. [LMK19017]*), motivation and self‐efficacy, a positive attitude towards treatment, willingness to compromise and pragmatism or cooperativity (*‘I'm just going to accept the situation as it is and try to make the best of it’ [BKHC2010]*).

*Symptoms* or lack thereof were almost solely reported by clinicians as facilitators. Thus, a lack of symptoms (good decisional capacity, communication and appearance, for e.g.) was seen as a vital prerequisite for SDM. Additionally, both patients and clinicians reported psychological strain to be a facilitating factor, as it motivated patient cooperation.

A good patient–clinician relationship, positive experiences with medication or the psychiatric facility and a patient's reasonable understanding of disease were also reported as facilitating factors.

#### 3.2.5 Facilitators of SDM—clinician‐related factors

*Characteristics/attitudes* (*n* = 27) and *behaviour during decisions* (*n* = 33) were the most commonly cited facilitators and stemmed mostly from patient interviews. Patients associated positive clinician traits with SDM, such as friendliness, pragmatism, conscientiousness, tolerance and helpfulness. Patients also saw specific attitudes as helpful: This included recognition of patients' wishes, taking the patient seriously, individual treatment and making an effort for their patients.

In terms of *clinicians' behaviour during decision making*, participants' reports included responding to patients' wishes/issues *(‘In talking to other patients I realized that [the doctors] really engage with everybody and aim to provide everyone with an individual package, so to speak, what he or she needs at that time […] I realized they go along with what the patients want and make it possible for them’. [LMK19017])*, readiness to compromise and the giving or receiving of feedback (*‘He always had the possibility to tell us he didn't agree with something and wanted to have a different way’. [BKHE2018‐dr]*)

Finally, a good *relationship with the clinician* was also considered to be important.

#### 3.2.6 Facilitators of SDM—setting‐related factors and others

*Setting‐related factors* included sufficient resources in terms of staff, an enjoyable atmosphere in the ward, the presence of other involved parties (nurses, other patients or legal guardians) and the nature of the decision. Thus, one patient reported that she would engage more in certain decisions, rather than others, as within the following example of a potential driving ban: *Patient: ‘If push comes to shove, then I think I would have to react like that. It sounds bad, and I don't want to go against another person's opinion. But I have to… my life and my independence are counting on this and… right now, this would be too big of a change’. (LMK19017)*

### 3.3 Co‐occurrence of paternalism and participation

What seemed remarkable to the researchers was the co‐occurrence of elements of paternalistic decision making and SDM. This was not only apparent within the entire pool of decisions (as shown in Figure 3) but also within the decisions of individual patient–clinician dyads, or even within a single specific decisional process. Thus, some decision descriptions contained elements of coercion or blackmail, while simultaneously incorporating elements of participation: *Physician: ‘The patient was always involved in all of these decisions. Like I said, she didn't accept the whole thing, because she didn't accept her diagnosis and was just willing to accept quetiapine; she was, however, repeatedly willing to take something after her privileges were taken away from her, so to speak, because of her poor condition, and she was then willing to accept the offered antipsychotics, but it was always her choice, which one it was’. (IAKBE015‐dr)*

## 4 DISCUSSION

### 4.1 Main findings

With the present qualitative analysis, we studied barriers and facilitators of SDM on acute psychiatric wards under ‘advantageous’ conditions, in which both patients and clinicians had received training in SDM, and more SDM had taken place compared to TAU.^8^ Even under these premises, a full range of stages of participation was present, ranging from coercion (no participation) to full participation. Our results indicate that usage of SDM with inpatients with schizophrenia is a complex matter and expressed by mostly preliminary stages of SDM. In addition, co‐occurrence of coercion and participation was observed. Finally, a number of SDM barriers and facilitators were identified.

### 4.2 Strengths and limitations

This study was preceded by a complex intervention that addressed both patients and clinicians, ultimately leading to an increased implementation of SDM in the intervention group.^8^ Researchers were able to choose interview partners from a large database and were thus able to select interviewees of different ages, sexes, disease severity and stages of acuity during the current hospital stay. Questioning both patients and their respective clinicians allowed for the comparison of perspectives of treatment decisions, that is, allowing for triangulation of the matter. An additional strength is the inclusion of patients who had been admitted involuntarily, since this vulnerable group had often been neglected in previous research.

One major limitation is that we did not observe the decision‐making process between clinicians and patients, but rather relied on participants' recollection of the event, which may at times have been biased by the presence of symptoms, or in some cases, by the patients' severe frustration with their situation (i.e., patients who had been brought into the facility against their will). On the other hand, some clinicians might have unintentionally overestimated their effort to increase patient participation. Additionally, the level model of participation was originally created within the context of citizens' political participation, and was thus not meant to describe the engagement of people with mental illness.

### 4.3 Comparison with previous literature

Existing literature reviews on barriers and facilitators to implementing SDM (e.g.^15^, ^16^) have already come up with various factors, often categorised as patient‐related factors, provider‐related factors and organisational factors, a similar categorisation as that found in our data. Most of the barriers found in previous studies were also present in our findings, including time constraints (lack of resources in our study), lack of applicability due to patient characteristics and the clinical situation.^16^ Additionally, qualitative studies that included mental health care providers have repeatedly highlighted consumers' lack of participation and communication problems as barriers to SDM.^17^, ^18^, ^19^ Especially patient characteristics and the clinical situation were also frequently cited in our study, with the main focus on psychotic symptoms and lack of insight. Symptoms may have been of special importance since our patient sample was markedly ill and hospitalised at the time of the interviews. Even during transcript analysis, symptoms were obvious in many cases. In terms of facilitators for SDM, Legare et al.^16^ name provider motivation, positive impact on the clinical process and patient outcomes. While provider motivation was also reflected in our data (‘attitude’), the other two factors were not present in our data.

However, several other facilitators were identified in our data set. Among these were hints on the importance of patient behaviour during consultations as a barrier and moreover as a facilitator for SDM. This issue has been rather neglected in the existing literature (except for e.g., Hamann et al.^9^ and Alegria et al.^9^, ^20^). It might be especially pronounced in our study, as active patient behaviour was one major goal of the SDM^PLUS^ intervention and patients in our sample predominantly expressed a more pronounced interest for participation in decision making compared to other samples.^21^ Likewise, the barrier/facilitator of physician behaviour might have been of importance in our data due to the behavioural focus of our intervention on the staff side (i.e., SDM, motivational interviewing, etc). The importance of both patients' and clinicians' behaviour in the decision‐making process as seen in our study is well in line with previous research findings,^22^, ^23^ which emphasised that communication is a vital element for SDM in clinician–patient interactions.

Finally, setting‐specific barriers (e.g., ward atmosphere or the fact that our interviews were mostly conducted on locked wards) have also been previously described.^6^, ^16^ Within these studies, the facilitating functions of trust, communication and information provision had also been highlighted. Contrary to the above‐cited studies,^6^ caregiver involvement was not considered as an important factor in our sample, either as a barrier or as a facilitator.

In our study, it was apparent that SDM elements were often combined with paternalistic elements. This could either be viewed as a way of embellishing an essentially paternalistic style of decision making or as a way of granting patients at least some form of participation. In several cases, however, more drastic measures including coercion and blackmail were reported. Similar research findings have summarised characteristics of this phenomenon as ‘informal coercion’.^24^

Finally, in mental health and also in the statements of the participants of our study, there are a variety of different decisions and the type of decision might have an influence on the decision‐making pattern. As in a previous study,^25^ drug decisions were very prominent in our sample. These rather medical decisions might, however, be made more paternalistically than, for example, psychosocial decisions, in which the patients might naturally have a greater say.^5^

### 4.4 Implications for clinical practice

We believe that there are at least three implications to SDM implementation, as derived from our analysis. These include a more thorough application of SDM interventions for patients and providers, the development of new approaches for specific patient needs (e.g., patients with mania) and the determination to implement as much SDM as is possible within the respective situation.

First, patients need to take active measures and therefore be regularly activated or empowered to facilitate SDM, which includes preparing for consultations and actively seeking clinicians, for example. Some patients already brought the necessary prerequisites to the consultation, including for example certain traits of character and a sufficient intrinsic motivation to participate. These attempts to facilitate joint decision making must be better acknowledged by clinicians to avoid patient frustration. Increased staff training in SDM,^26^ probably best beginning during early medical training, might be helpful to better implement SDM strategies.^27^ Other patients might not be as well equipped for SDM and may need more support. This could be achieved by patient groups such as the one offered in the SDM^PLUS^ trial, which aims to increase patient engagement while simultaneously requiring clinicians to encourage patient participation.

Second, SDM and additional related measures (such as motivational interviewing) might still not help to engage 100% of patients in joint decision‐making processes. Two symptom complexes are to be named here: Acute mania, which limits the possibility of effective conversations, and negative symptoms, which can include extreme passiveness and lack of drive. As stated above, clinicians must proactively address negative symptoms. However, the study was not able to provide a concrete proposal for solution of manic symptoms. One approach could include a delayed use of SDM at later stages of the disease, when some patients might be better able to engage in decision making.

Finally, our study suggests that a combination of SDM and paternalism in some is a more effective strategy than pure paternalism. Thus, we would propose an ‘as much SDM as possible’ strategy, which would encourage the use of SDM in every situation.^28^

## 5 CONCLUSION

Even in the context of a preceding successful SDM intervention, the implementation of SDM for patients in the very acute stages of schizophrenia is often not possible. However, physicians attempting to engage patients to a greater extent may already be appreciated by patients and lead to higher perceived involvement. Beneath barriers, strong facilitators for SDM have also been identified, which should be used for further implementation of SDM.

## CONFLICT OF INTERESTS

Dr. Hamann reports grants from Janssen Cilag, Germany, during the conduct of the study, personal fees from Janssen Cilag, Germany, personal fees from Otsuka, and personal fees from Lundbeck, outside the submitted work. In the past three years Stephan Heres has accepted speaker honoraria from Johnson & Johnson and Otsuka/Lundbeck; services as consultant in clinical trials were provided for the companies TEVA, ROVI and KYE. Mrs. Becher and Dr. Holzhüter have nothing to disclose. [Correction added on 28 July 2021, after first online publication: Stephan Heres declares conflict of interests].

## Supporting information

Supporting information.

## Data Availability

Data available on request due to privacy/ethical restrictions.
