# Supplementary material for: Barriers and facilitators of shared decision making in acutely ill inpatients with schizophrenia—Qualitative findings from the intervention group of a randomised‐controlled trial
Source: Health Expect. 2021 Jul 13;24(5):1737–46. doi: 10.1111/hex.13313 (PMC8483208; doi:10.1111/hex.13313)
Supplement: Supplementary file 1 — Supporting information. [file HEX-24-1737-s001.docx]

Interview guide:

Patients:

| Main topics | Possible prompts |
| --- | --- |
| - - - *Please tell me about your satisfaction with treatment here in the hospital* | - - - - Differences between start to end? |
| - - - *Please tell me about how you were able to participate in decision making during the inpatient stay* | - - - - Differences between start to end? |
| - - - *How did you experience consultations with your clinicians?* | - - - - Accessibility?       - Initiation through whom?       - Course of the consultation?       - Duration?       - Decisions discussed? |
| - - - *Can you tell me more about a specific decision that has been made during your inpatient stay?* | - - - - What was the decision about?       - How did the decision take place?       - Initiator of the decision?       - Level of participation during the decision?       - Level of satisfaction with the decision process?       - Suggestions for improvement? |
| - - - Additional remarks regarding… | - - - Patient group “How to talk to your psychiatrist”     - Leave regulations     - Coercive measures |

Clinicians:

| Main topics | Possible prompts |
| --- | --- |
| - - - *How was the treatment course of this patient and how was your relationship with the patient?* | - - - - Differences between start to end? |
| - - - *Please tell me about how the patient participated in decision making during the inpatient stay* | - - - - Differences between start to end? |
| - - - *How did you experience consultations with your patient?* | - - - - Differences between start to end? |
| - - - *Can you tell me more about a specific decision that has been made during the inpatient stay of this patient?* | - - - - What was the decision about?       - How did the decision take place?       - Initiator of the decision?       - Level of participation during the decision?       - Suggestions for improvement?       - Barriers and facilitators towards SDM |
| - - - Additional remarks regarding… | - - - Patient group “How to talk to your psychiatrist”     - Leave regulations     - Coercive measures |
